# Electroacoustic Comparison of Hearing Aid Output of Phonemes in Running Speech versus Isolation: Implications for Aided Cortical Auditory Evoked Potentials Testing

**DOI:** 10.1155/2012/518202

**Published:** 2012-12-18

**Authors:** Vijayalakshmi Easwar, David W. Purcell, Susan D. Scollie

**Affiliations:** ^1^Health and Rehabilitation Sciences (Hearing Sciences), National Centre for Audiology, Faculty of Health Sciences, Western University, London, ON, Canada N6G 1H1; ^2^National Centre for Audiology and School of Communication Sciences and Disorders, Western University, London, ON, Canada N6G 1H1

## Abstract

*Background*. Functioning of nonlinear hearing aids varies with characteristics of input stimuli. In the past decade, aided speech evoked cortical auditory evoked potentials (CAEPs) have been proposed for validation of hearing aid fittings. However, unlike in running speech, phonemes presented as stimuli during CAEP testing are preceded by silent intervals of over one second. Hence, the present study aimed to compare if hearing aids process phonemes similarly in running speech and in CAEP testing contexts. *Method*. A sample of ten hearing aids was used. Overall phoneme level and phoneme onset level of eight phonemes in both contexts were compared at three input levels representing conversational speech levels. *Results*. Differences of over 3 dB between the two contexts were noted in one-fourth of the observations measuring overall phoneme levels and in one-third of the observations measuring phoneme onset level. In a majority of these differences, output levels of phonemes were higher in the running speech context. These differences varied across hearing aids. *Conclusion*. Lower output levels in the isolation context may have implications for calibration and estimation of audibility based on CAEPs. The variability across hearing aids observed could make it challenging to predict differences on an individual basis.

## 1. Introduction

Hearing aid validation using aided speech evoked auditory evoked potentials is of research and clinical interest. Such measurements involve elicitation of an evoked potential using a speech stimulus that has been processed through a hearing aid. Hearing aids, being mostly nonlinear, may have implications for the nature of speech stimulus used as input. The present study focuses on the effect of nonlinear hearing aid processing on speech stimuli used for measurement of cortical auditory evoked potentials (CAEPs). 

Nonlinear hearing aids are sensitive to the characteristics of input stimuli. Factors such as input level, duration, crest factor (ratio of peak to root mean square (RMS) amplitude), modulation depth, and modulation frequency of the input signal may affect the gain applied by the hearing aid, in ways that would not occur with a linear system [1–4]. These effects have been attributed to the level-dependent signal processing architecture, which in many hearing aids includes frequency specific compression threshold, compression ratio, compression time constants, number of channels, gain in each channel, expansion threshold, and expansion time constants [1, 5–12]. In addition, hearing aid processing may also consider the frequency characteristics of the input stimulus (e.g., [13, 14]). Hence the output of a hearing aid to a specific input is the product of complex interactions between input stimuli and hearing aid features that may or may not be known to or may not be adjustable by the end user. 

Nonlinear hearing aids, being sensitive to features of the input signal, process speech or speech-like stimuli differently from nonspeech stimuli [3, 7, 10, 15]. Since the main goal of hearing aid validation procedures is to assess benefit of hearing aid use while listening to speech, it is preferable that such procedures use speech stimuli in the most natural or frequently encountered form as possible. Behavioural validation procedures (tests that require active participation of the hearing aid user) such as speech tests, mostly use speech in various natural forms. Examples include the use of sentence materials, such as the Bamford-Kowal-Bench sentence test [16], or materials with less grammatical context such as isolated words or nonsense syllables (e.g., The Nonsense Syllable test [17]). But the speech stimuli may need to be modified for use in alternative validation methods such as aided auditory evoked potentials [18–23]. 

Aided auditory evoked potentials are objective and electrophysiological (they record neural responses to sound) but historically have not used speech stimuli. Of these, one of the reasons CAEPs have been of interest in the validation of hearing aid fittings is because natural speech sounds can be used as stimuli [19, 23–27]. Often phonemes or syllables excised from running speech or from standard speech tests have been used to record reliable CAEPs (e.g., [27–29]). Although natural speech can be used as stimuli, CAEP testing involves presentation of these stimuli with interstimulus intervals (ISI). These ISIs usually range on the order of 1-2 seconds (e.g., [23, 29, 30]) optimized for the latency of CAEPs and refractory periods of the cortical pyramidal neurons [30–32]. These stimuli are repeated 100–200 times, with constant or slightly variable ISIs and CAEPs elicited to each of the presentations are averaged. Presence of a CAEP elicited by a specific stimulus is interpreted as the stimulus being relayed to the source of CAEPs, the auditory cortex [21, 24]. Evidence suggests that CAEP thresholds (i.e., the lowest stimulus level at which a CAEP is detected) are closely related to behavioral thresholds (i.e., the lowest stimulus level at which the participant detects the stimulus) [33, 34]. Therefore, presence of a CAEP is likely to suggest audibility of the eliciting stimulus. On these premises, recent aided CAEP protocols for hearing aid validation have used brief segments of speech in the form of phonemes or syllables (e.g., [21–25]). Depending on their length, these brief segments may differ in their representation of certain features cues such as formant transitions, compared to longer segments of these same phonemes embedded in running speech. Commercial equipment such as the HEARLab uses phonemes, sampled across the speech frequency range presented at their naturally occurring levels within running speech, and presented in isolation to permit averaging of CAEP across several sweeps [35]. 

Phonemes presented in isolation for CAEP protocols may differ in several important ways from phonemes presented within running speech. In CAEP protocols, the target phoneme is preceded by an ISI (a silence period) whereas the same phoneme in running speech is likely to be preceded by other phonemes. Since nonlinear hearing aids continuously and rapidly adjust band-specific gains based on the acoustic input, there is a possibility that the hearing aids may react differently to the same phoneme when presented during aided CAEP testing as compared to when they occur in running speech. With 1-2 seconds of ISI preceding every repetition of the stimulus, nonlinear hearing aids may demonstrate an overshoot at the onset of the stimulus consistent with compression circuitry [36]. Also, hearing aids of different models and different manufacturers may vary in how quickly they respond to changes in the acoustic input. Therefore, verifying that hearing aid output is comparable for phonemes presented in these two contexts (preceding silent periods/ISI versus embedded in running speech) may be an important step in evaluating the validity of using CAEP protocols in hearing aid validation. Previous reports on non-CAEP related measures suggest that certain features of nonlinear signal processing in hearing aids may attenuate the level of speech sounds immediately preceded by silence [37, 38]. 

The effects of CAEP protocols on the gain achieved while processing tone bursts have been reported elsewhere in this issue [40, 41]. These studies provide evidence that hearing aid gain differs for tone bursts (short and long) presented in isolation versus pure tones that are continuous. Specifically, the gain achieved during processing of tone bursts was lower than the verified gain, when measured at 30 ms poststimulus onset and at maximum amplitude. Onset level is of interest because the first 30 to 50 ms of the stimulus primarily determines the characteristics of the elicited CAEP [42]. Stimulus level of the hearing aid processed tone bursts was positively related to the CAEP amplitude, with stimulus level at 30 ms poststimulus onset being a better predictor of CAEP amplitude compared to maximum stimulus level. These reports [40, 41] substantiate the need to verify output levels of CAEP stimuli across contexts, and to consider stimulus onsets. The present study will focus upon aided processing of phonemes across contexts and measure both overall level (level measured across the entire duration of the phoneme) and onset level of the stimuli at the output of the hearing aid. 

The purpose of this study was to understand if hearing aids process CAEP phonemes presented in isolation differently to phonemes presented in running speech. The primary outcome measure of interest in this study was the output level of phonemes in both contexts. Findings from this study may provide some insights into the design of hearing aid validation protocols that employ aided CAEP measures, because large differences in hearing aid output arising due to stimulus context may influence interpretation of audibility based on aided CAEPs.

## 2. Method

### 2.1. Hearing Aids

Ten hearing aids sampled across various manufacturers were chosen. A list of the hearing aids used is provided in Table 1. Hearing aids were sampled across a representative range of major manufacturers and were behind-the-ear (BTE) in style. Of the 10 hearing aids, six were programmed and verified to meet DSL v5a adult prescription targets [43] for an N4 audiogram [39]. The N4 audiogram represents hearing loss of moderate to severe degree with thresholds of 55 dB HL at 250 Hz worsening down to 80 dB HL at 6 kHz [39]. The remaining four hearing aids were programmed and verified to meet DSL v5a targets for an N6 audiogram. The N6 audiogram represents hearing loss of severe degree with thresholds ranging from 75 dB HL at 250 Hz worsening to 100 dB HL at 6 kHz [39]. The frequency specific thresholds of the two audiograms used are provided in Table 2. Hearing aids appropriate for different audiograms were chosen from different manufacturers to obtain a representative sample of commonly available commercial products. All hearing aids were programmed to function on a basic program with all additional features such as noise reduction, feedback cancellation, and frequency lowering disabled during verification and recording. As such, variance across devices is mainly attributable to the nonlinear characteristics of the devices, in isolation of these other aspects of hearing aid signal processing.

### 2.2. Stimuli

Stimuli were constructed to have both running speech and phoneme-in-isolation contexts as follows. For the running speech context, eight phonemes (/a/, /i/, /u/, /s/, /*∫*/, /m/, /t/, and /g/) were identified within a recording of the Rainbow passage. The passage was spoken by a male talker and lasted 2 minutes and 14 seconds. Aided recordings of this passage were made for each hearing aid, and the level of each phoneme was measured from within the aided passage. For the isolated context, the same phonemes and phoneme boundaries were used, but were excised from the passage for use as individual stimuli. Boundaries of these phonemes were chosen such that any transitions preceding and following these phonemes due to coarticulation were excluded. The duration of each of the phonemes are as follows: /a/—87 ms, /i/—84 ms, /u/—124 ms, /s/—133 ms, /*∫*/—116 ms, /m/—64 ms, /t/—26 ms, and /g/—19 ms. The durations of these phonemes differed naturally and were not modified in order to allow direct comparisons between the two contexts. These specific phonemes were chosen as the first six of these phonemes are a part of the commonly used Ling 5 or 6 sounds test [44, 45]. The last three have been commonly used in a series of aided CAEP studies (e.g., [26, 27, 46]) and are also a part of the stimulus choices available in the HEARLab [35]. A silent interval of 1125 ms preceding each phoneme was created using sound editing software Goldwave (v.5.58). This is to simulate a CAEP stimulus presentation protocol where the ISI usually ranges between one and two seconds. 

### 2.3. Recording Apparatus

Recordings of hearing aid output used a click-on coupler (Brüel & Kjær (B&K) type 4946 conforming to ANSI S3.7, IEC 60126 fitted with microphone type 4192) with an earplug simulator. The hearing aid was connected via 25 mm of size 13 tubing [47]. This was set up in a B&K anechoic box (Box 4232) that also housed a reference microphone. Stimuli were presented through the speaker housed in the box. The outputs of the reference and coupler microphones were captured in SpectraPLUS (v5.0.26.0) in separate channels using a sampling rate of 44.1 kHz with 16-bit sampling precision. SpectraPLUS software was used to record the reference and coupler signals as  .wav files for further signal analyses. 

### 2.4. Recording Procedure

Running speech was presented at overall RMS levels of 55, 65, and 75 dB SPL. These levels approximate speech at casual through loud vocal effort levels [48]. Since individual phonemes naturally varied in their relative levels within the Rainbow passage, the level of each isolated phoneme was matched to the level at which it occurred in the Rainbow passage, for each presentation level. With this recording paradigm, the overall input levels of each phoneme were matched between the two contexts. During presentation of phonemes in the isolation context, approximately 10 repetitions of each phoneme (each preceded by ISI of 1125 ms) were presented during any single recording. 

### 2.5. Output Measures

**Figure 1:**
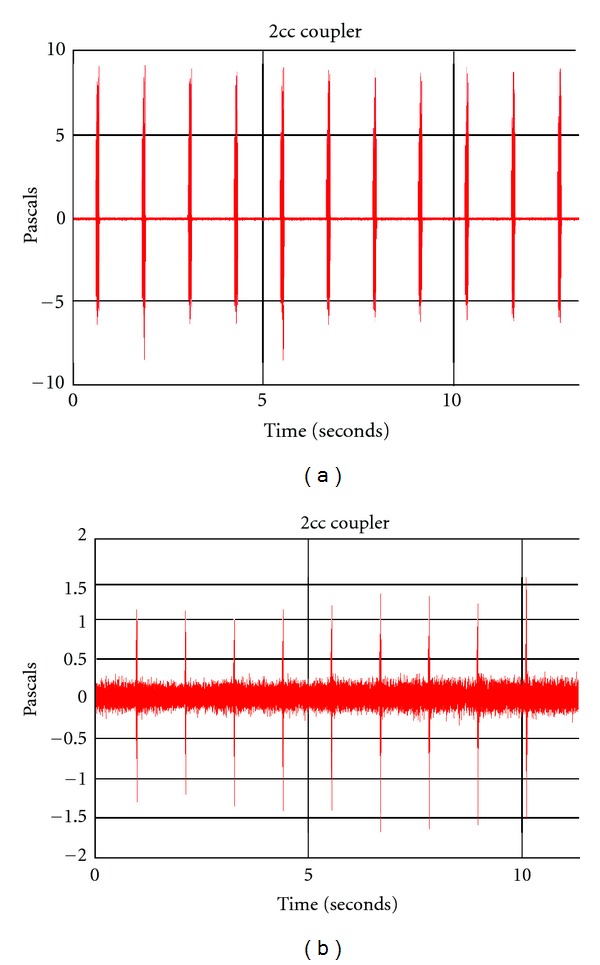
(a) illustrates the amplitude-time waveform of the output of one of the hearing aids when the stimulus /a/ was presented at 65 dB SPL. The hearing aid was programmed to DSL v5 targets derived for the audiogram N4. The first few repetitions are more variable than the later repetitions. (b) illustrates the amplitude-time waveform of the output of one of the hearing aids when the stimulus /g/ was presented at 55 dB SPL. The hearing aid was programmed to DSL v5 targets derived for the audiogram N4. The first few repetitions are lower in level compared to the later repetitions.

Measurements were carried out offline using SpectraPLUS. Two measurements were made per phoneme and per context: the overall level of the phoneme (dB SPL RMS recorded over the entire duration of the phoneme) and the onset level of the phoneme (dB SPL RMS recorded over the first 30 ms of the stimulus phoneme). Onset measurements could not be completed for phonemes /t/ and /g/ as the duration of these phonemes was shorter than 30 ms. For these phonemes, we therefore report only overall phoneme levels. In the isolation context, measurements were completed after the first few repetitions of the phoneme. The first few repetitions were discarded as, in our preliminary recordings using a few hearing aids, interrepetition variability was observed to be high in the first few repetitions. This is likely related to nonlinear signal processing in the hearing aids but these effects were not formally evaluated in this study. Figures 1(a) and 1(b) illustrate examples of the variability observed in the first few repetitions.

### 2.6. Analyses

Repeated measures of analysis of variance (RM-ANOVA) were completed using SPSS (v. 16) with context (running speech and isolation), level (55, 65, and 75 dB SPL), and phoneme as the three independent factors. Separate analyses were carried out for overall phoneme level and onset level. Greenhouse-Geisser corrected degrees of freedom were used for interpretation of all tests. Multiple paired *t*-tests were completed to explore significant context interactions. For interpretation of these multiple *t*-tests, sequential Bonferroni type corrections that control for false discovery rates were used to determine critical *P* values [49, 50]. 

## 3. Results 

Phonemes embedded in running speech were measurable for nearly all hearing aids in this study. For one of the hearing aids, the output level of /g/ in isolation at 55 dB SPL input level could not be measured as it was embedded within the hearing aid noise floor. Across the sample, the average overall phoneme level measured in the running speech context was 94.07 dB SPL (standard error (SE) = 1.79 dB) and in the isolation context was 92.43 dB SPL (SE = 1.94 dB). On average, the phoneme onset level measured in the running speech context was 94.67 dB SPL (SE = 1.79 dB) and in the isolation context was 94.44 dB SPL (SE = 1.83 dB). The outcome of statistical tests for overall phoneme level and phoneme onset level will be described below. 

### 3.1. Difference in Overall Phoneme Level across Contexts

**Figure 2:**
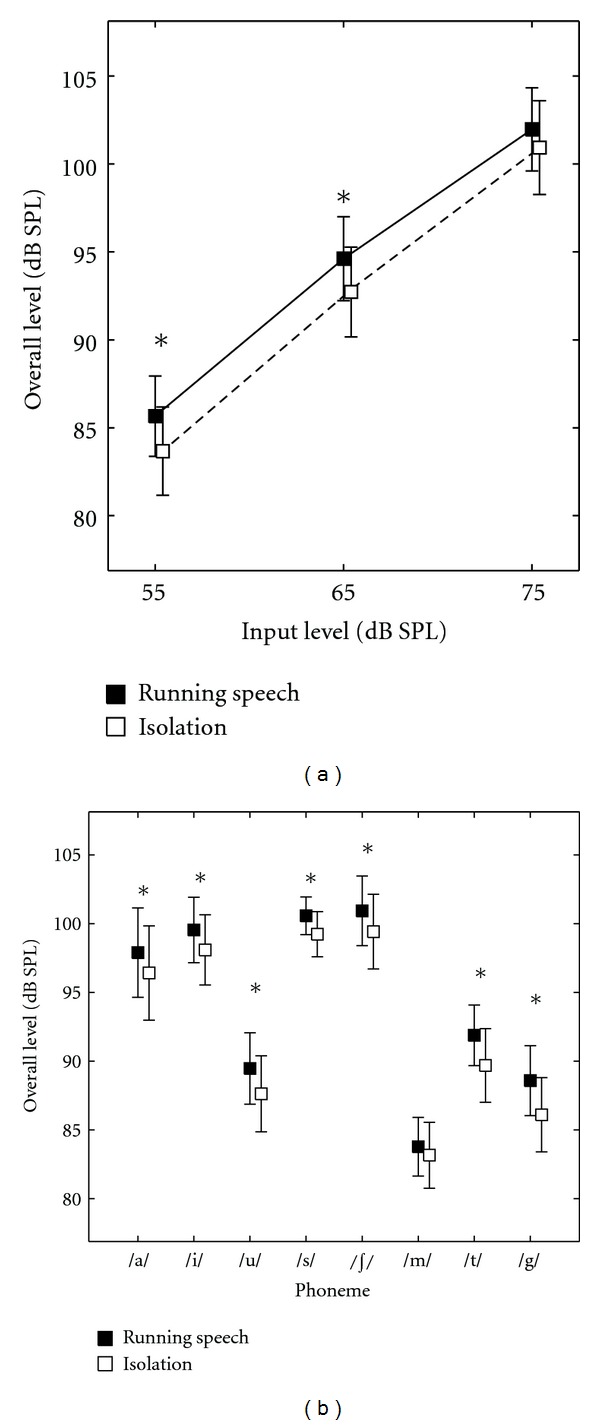
(a) presents variation of overall phoneme level in running speech and isolation context across input levels. (b) presents the same across phonemes. Error bars represent SE. ∗ indicates a statistically significant difference in paired contrasts. The symbols have been offset slightly to improve clarity.

RM-ANOVA revealed a significant effect of context (*F* = 10.114 [1, 8], *P* = 0.013), input level (*F* = 834.58 [1.02, 8.12], *P* < 0.001), and phoneme (*F* = 93.26 [1.95, 15.62], *P* < 0.001). Interactions between input level and context (*F* = 8.36 [1.35, 10.82], *P* = 0.011), phoneme and context (*F* = 3.38 [2.63, 21.05], *P* = 0.042), and input level and phoneme (*F* = 5.25 [2.69, 21.56], *P* = 0.009) were also significant. The three-way interaction between input level, context, and phoneme was not significant (*F* = 1.061 [2.48, 29.79], *P* = 0.388). Paired contrasts comparing overall phoneme levels between contexts at each input level showed significant differences at the 55 and 65 dB SPL input levels but not at the 75 dB SPL input level. At input levels of 55 and 65 dB SPL, the levels of phonemes were significantly higher when they appeared in running speech compared to when they occurred in isolation (see Figure 2(a) and Table 3 for group means). In summary, the difference between contexts reduced as input level increased. 

Paired contrasts comparing overall phoneme levels between contexts for each phoneme showed significant differences for all phonemes except /m/ (see Figure 2(b) and Table 4 for group means). All phonemes except /m/ were higher in level when they occurred in running speech compared to when they occurred in isolation. 

### 3.2. Difference in Phoneme Onset Level across Contexts

**Figure 3:**
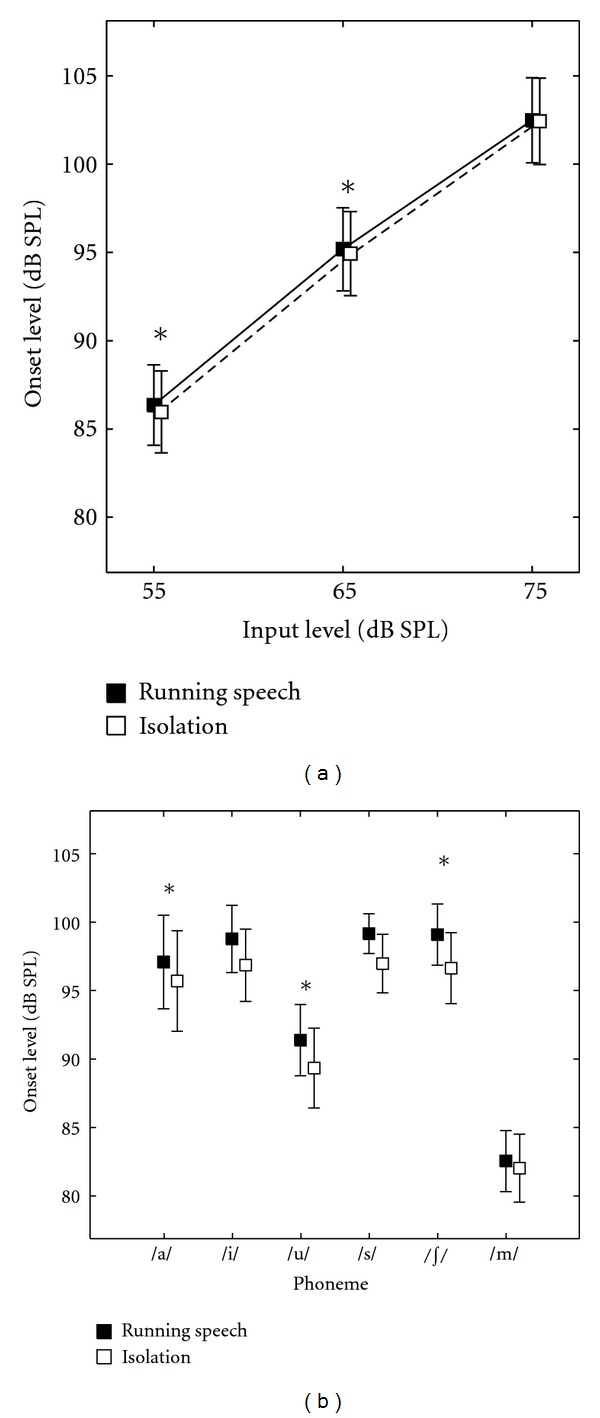
(a) presents variation of phoneme onset level in running speech and isolation context across input levels. (b) presents the same across phonemes. Error bars represent SE. ∗ indicates a statistically significant difference in paired contrasts. The symbols have been offset slightly to improve clarity.

A similar result was obtained for phoneme onset level. RM-ANOVA revealed a significant effect of context (*F* = 7.41 [1, 9], *P* = 0.024), input level (846.94 [1.05, 9.44], *P* < 0.001), and phoneme (*F* = 52.84 [1.78, 16.04], *P* < 0.001). Interactions between input level and context (*F* = 17.71 [1.20, 10.81], *P* = 0.001), and phoneme and context (3.95 [3.45, 31.09], *P* = 0.013) were significant. Interaction between input level and phoneme (*F* = 1.49 [2.06, 18.56], *P* = 0.250) and the three-way interaction between input level, context, and phoneme were not significant (*F* = 0.89 [3.25, 29.25], *P* = 0.473). Paired contrasts between phoneme onset levels of both contexts at each input level showed significant differences between contexts at 55 and 65 dB SPL but not at the 75 dB SPL input level. At input levels of 55 and 65 dB SPL, the onset levels of phonemes were significantly higher when they appeared in running speech compared to when they occurred in isolation (see Figure 3(a) and Table 3 for group means). Similar to overall phoneme level, the difference between contexts reduced with increasing input level. 

Paired contrasts comparing phoneme onset levels between contexts for each phoneme revealed no significant differences for all phonemes except /*∫*/ and /u/ (see Figure 3(b) and Table 4 for group means). Phonemes /*∫*/ and /u/ were higher in onset level when they occurred in running speech compared to when they occurred in isolation. 

### 3.3. Individual Differences across Hearing Aids

The mean difference in overall phoneme level averaged across hearing aids, input levels, and phonemes was found to be 1.64 dB, where phonemes in running speech measured higher on average. The mean difference in phoneme onset level computed similarly was 0.23 dB, onset of phonemes in running speech measuring higher on average. Although the mean value suggests a clinically insignificant difference due to context, inspection of individual data highlights the differences observed across hearing aids and phonemes. Tables 5(a) and 5(b) provide the difference (in dB) in the output measures (overall phoneme level and phoneme onset level) in both contexts, averaged across all three input levels. These differences were obtained by subtracting the level of each phoneme in isolation from the corresponding level in running speech. Hence, a positive value indicates that the level of the phoneme is higher when it occurs in running speech, as it would in daily life, versus in isolation, as it would during CAEP measurement. Differences of greater than 3 dB are presented in bold. 

The proportion of difference values greater than ±3 and ±5 dB are presented in Table 6 for both overall phoneme levels and phoneme onset levels at each input level. Pooled across both directions of differences and input levels, about 24% of the overall phoneme levels (total of 239 observations across three levels, 10 hearing aids and eight phonemes, 1 missing value) showed differences of greater than ±3 dB and 7% showed differences of greater than ±5 dB. In case of phoneme onset levels, about 33% of the observations (total of 180 observations across three levels, 10 hearing aids and six phonemes) showed differences of over ±3 dB and nearly 13% showed differences of over ±5 dB. In general, differences greater than 3 dB are well outside of test-retest differences in electroacoustic measurement, while differences greater than 5 dB are greater than a typical audiometric step size. The latter is likely clinically significant, while the former may have impact for interpretation of research data and calibration. We note that the majority of aided phoneme levels agreed between the two contexts within ±3 dB.

## 4. Discussion 

Results suggest that hearing aid output level of a phoneme in isolation may either match or may differ from the output level of the same phoneme when it occurs in running speech. Agreement was observed in approximately 66% to 75% of cases, while differences exceeding 3 dB were observed in 24% to 33% of cases. Agreement occurred in more cases (75%) for measures of overall level of phoneme, and in fewer cases (66%) for measures of phoneme onset level. When differences existed, they typically manifested as the hearing aid producing a lower output for the phoneme in isolation than it did for the phoneme in running speech. Differences reduced with increases in input level and varied across phonemes and hearing aids. Similar trends were observed in overall phoneme level and phoneme onset level. 

Results from the present study are similar to the findings from other reports in this issue [40, 41]. Specifically, these reports and the current study show that across measurement strategies and stimulus types, hearing aids may apply lower gain and output (at onset as well as at maximum amplitude) to brief stimuli that are immediately preceded by silence, such as those commonly used to elicit the CAEP. However, one may note that the hearing aids used in these studies [40, 41] were set to function linearly, unlike the hearing aids used in the present study. Another study has used a nonlinear hearing aid to study the effect of hearing aid processing on the tone burst onset while comparing it with the unaided condition [36]. The aided condition in this study produced a marginal increase in the level at onset due to the presence of an overshoot. In the present study, there were fewer instances of significant overshoot, but recall that the unaided condition was not assessed in this study. Therefore, the present results pertain only to the comparison of aided levels between the isolation context and running speech. Overshoot may be present in both conditions. Also, the effects of overshoot attributable to nonlinear signal processing in hearing aids may vary across devices, with the effects being idiosyncratic to specific devices or stimuli. Results similar to the majority of the observations in the present study have also been noted in non-CAEP related studies of nonlinear signal processing in hearing aids [37, 38]. 

### 4.1. Effect of Input Level and Phoneme on Difference due to Context

The decrease in differences in overall and onset level of phonemes between contexts with increase in input level could indicate an effect of output limiting. As the output levels of phonemes come close to the maximum power output of the hearing aids, they are subject to compression limiting [1, 5]. Compression limiting restricts the maximum output level by using a very high or infinite compression ratio in an output controlled compression system [1]. Hence, at higher input levels, where the output levels are likely subject to output limiting in both stimulus contexts, the differences seen are smaller compared to lower input levels that are relatively less likely to be affected by output limiting. 

Analyses revealed that differences across contexts varied across phonemes. We did not perform a direct comparison across phonemes because the individual phonemes occur at different levels relative to, the overall RMS level of running speech. Compression, being a level-dependent nonlinear factor in the hearing aid, may therefore vary the gain applied for each of these phonemes, especially when they are presented in isolation. In addition, compression features such as compression ratio and time constants were likely different across different frequencies due to the slightly sloping configurations of audiograms chosen and the presence of multiple channels in our hearing aid sample. Since phonemes varied in their spectral composition and position of spectral peaks, they could have been subject to different compression features in different channels. One stimulus characteristic that could have been influential in determining overall phoneme output levels is the duration of phonemes.  Table 5(a) suggests that differences larger than 3 dB occurred more often for /g/ and /t/ relative to other phonemes. Among all eight phonemes, /t/ and /g/ were the lowest in level and shortest in duration, measuring 26 ms and 19 ms, respectively. This may have made these phonemes in isolation more susceptible to the dynamic effects of hearing aid nonlinearity [1, 37, 38]. However, this study did not study systematically the effects of duration and level as they interact with context. Further study on this may be necessary to determine the effects of phoneme level and duration. Also, the preceding context within running speech may have differed in ways crucial to determination of gain/compression characteristics for the target phoneme. 

### 4.2. Interhearing Aid Variability

Tables 5(a) and 5(b) illustrate that individual hearing aids may amplify individual phonemes differently, even though they were set to produce similar gain for long-duration signals. Hearing aids not only varied in differences due to context but also showed differences for the same phoneme in the same context. This illustrates that different manufacturers may employ different nonlinear signal processing strategies. Differences across hearing aid manufacturers were also reported by Jenstad et al. [40]. Differences in other parameters across hearing aid manufacturers have also been reported among hearing aids that were matched in gain characteristics (e.g., sound quality comparisons by Dillon et al. [51]). The finding that hearing aids show large individual variability makes it challenging to predict the nature of differences on a case-by-case basis in clinical practice.

### 4.3. Implications for Aided CAEP Testing

CAEPs are level dependent [26, 46, 52, 53]. Parameters such as amplitude and latency of individual peaks reflect changes in stimulus level or sensation level of the stimulus with reference to the behavioral threshold of the CAEP stimulus. A change in sensation level of the stimulus from a positive (above threshold; audible) to a negative (below threshold; inaudible) value is likely to decrease the probability of eliciting a CAEP. If output levels of phonemes in running speech are considered to be the reference condition of interest, CAEP test measures may underestimate audibility when phonemes are presented in isolation. These data indicate that underestimation is minimal (about 2 dB) on average, but was between 3 and 8 dB in over 24% of cases. There were also instances that may result in overestimation of audibility, but these are far fewer in number and magnitude. 

Since the experimental conditions used in this study were limited to one duration of ISI and one naturally occurring preceding context per phoneme, generalization to other instances and variation across durations or levels of phonemes may require further investigation. Investigation of the effects of hearing aid signal processing on spectral characteristics such as formant transitions may also be possible, but these effects were not evaluated in this study. The effects of other aspects of hearing aid signal processing, such as digital noise reduction, may also be relevant and were not explored in this study. Based on this study, we conclude that significant differences in hearing aid functioning between running speech and isolated phoneme contexts occur, along with considerable interhearing aid variability. In over a fourth of aided phonemes, the magnitude of these differences was large enough to impact calibration, or interpretation of group data. This may indicate the need to perform acoustic calibration for individual hearing aids for the purpose of well-defined CAEP stimuli. In 7%–13% of phonemes, the differences exceeded that of an audiometric step size and therefore may be clinically important. 

## Figures and Tables

**Table 1:** 

Hearing aids for N4 audiogram	Hearing aids for N6 audiogram
*Oticon Agil Pro P *	*Oticon Chilli SP *
*Phonak Nios Micro V *	*Phonak Naida IX SP *
*Siemens Aquaris 701 *	*Unitron 360+ *
*Widex Mind 330 *	*Starkey S series IQ 11 *
*Unitron Passport *	
*Siemens Motion 701p *	

**Table 2:** Frequency specific thresholds of N4 and N6 standard audiograms [39]. The threshold at 750 Hz for the N6 audiogram was originally 82.5 dB HL but had to be rounded to 85 dB HL to allow input into the verification system.

Audiogram	Frequency specific thresholds (dB HL)								
	250	500	750	1 kHz	1.5 kHz	2 kHz	3 kHz	4 kHz	6 kHz
N4	55	55	55	55	60	65	70	75	80
N6	75	80	85	85	90	90	95	100	100

**Table 3:** Results of post hoc tests for level context interaction.

	Input level	Running speech (mean (dB SPL), SE (dB))	Isolation (mean (dB SPL), SE (dB))	*t*-statistic, df	*P* value	Critical *P* value
	55	85.66, 2.28	83.66, 2.51	4.437, 9	0.002*	0.017
Overall level	65	94.61, 2.38	92.72, 2.55	3.803, 9	0.004*	0.033
	75	101.97, 2.36	100.9, 2.66	1.173, 9	0.121	0.050

	55	86.35, 2.27	85.96, 2.31	4.234, 9	0.002*	0.017
Onset level	65	95.18, 2.35	94.93, 2.38	2.739, 9	0.023*	0.033
	76	102.48, 2.41	102.43, 2.44	0.446, 9	0.653	0.050

*Indicates a statistically significant difference.

**Table 4:** Results of post hoc tests for phoneme context interaction.

	Phoneme	Running speech (mean (dB SPL), SE (dB))	Isolation (mean (dB SPL), SE (dB))	*t*-statistic, df	*P* value	Critical *P* value
Overall level	/a/	97.89, 3.25	96.41, 3.43	3.197, 9	0.011*	0.025
	/i/	99.54, 2.38	98.09, 2.55	2.856, 9	0.019*	0.031
	/u/	89.46, 2.59	87.62, 2.77	4.231, 9	0.002*	0.006
	/s/	100.57, 1.37	99.23, 1.64	3.506, 9	0.007*	0.019
	/*∫*/	100.93, 2.52	99.42, 2.71	3.981, 9	0.003*	0.013
	/m/	83.77, 2.13	83.16, 2.39	0.954, 9	0.365	0.050
	/t/	91.88, 2.20	89.69, 2.67	2.425, 9	0.038*	0.044
	/g/	88.57, 2.54	88.1, 2.70	2.450, 9	0.037*	0.038

Onset level	/a/	97.09, 3.41	95.69, 3.67	2.376, 9	0.042	0.042
	/i/	98.77, 2.45	96.85, 2.64	2.588, 9	0.029	0.025
	/u/	91.37, 2.60	89.34, 2.91	3.143, 9	0.012*	0.017
	/s/	99.16, 1.44	96.97, 2.14	2.497, 9	0.034	0.033
	/*∫*/	99.08, 2.24	96.63, 2.59	4.161, 9	0.002*	0.008
	/m/	82.54, 2.23	82.07, 2.48	0.634, 9	0.542	0.050

*Indicates a statistically significant difference.

**Table:** (a)

Hearing aid	Phoneme							
	/a/	/i/	/u/	/s/	/*∫*/	/m/	/t/	/g/
1	0.68	0.56	**3.88**	2.66	**3.47**	1.58	2.45	2.25
2	**3.10**	2.53	**3.40**	1.16	2.16	**3.27**	**6.54**	**6.03**
3	2.46	**3.58**	**3.45**	2.89	2.29	2.20	**5.15**	**6.23**
4	**4.56**	**4.72**	1.84	**3.22**	**3.24**	**3.44**	**5.23**	**4.69**
5	−0.31	−0.01	−0.06	0.85	0.29	−2.20	−1.28	−0.46
6	1.19	0.55	0.75	0.40	0.61	0.44	2.60	1.50
7	0.62	0.70	1.73	0.60	0.59	−1.09	−1.05	0.77
8	0.62	0.92	1.46	1.55	1.34	0.31	**3.00**	**5.22**
9	0.70	0.25	1.85	0.53	0.42	0.34	−0.12	**3.66**
10	1.15	0.65	0.13	−0.43	0.67	−2.16	−0.61	−0.39

**Table:** (b)

Hearing aid	Phoneme					
	/a/	/i/	/u/	/s/	/*∫*/	/m/
1	−0.39	0.01	2.53	2.23	**3.64**	0.52
2	2.44	2.81	**3.69**	1.81	**3.74**	**3.95**
3	**4.11**	**6.53**	**6.81**	**7.06**	**5.69**	**3.04**
4	**4.70**	**5.37**	2.28	**7.48**	**4.45**	**4.31**
5	−0.47	−0.71	−0.43	0.71	1.37	−2.59
6	1.72	0.70	1.05	0.05	1.26	−0.38
7	0.12	0.84	0.99	0.29	0.66	−2.23
8	−0.17	1.78	2.06	1.27	2.67	0.81
9	0.55	0.51	1.25	0.34	0.82	0.38
10	1.33	1.36	0.19	0.61	0.19	−2.61

**Table 6:** Proportion of observations (%) showing differences greater than 3 or 5 dB (positive value indicates higher output levels of phoneme in running speech).

	Input level	>3 dB	<−3 dB	>5 dB	<−5 dB
	55	9.62	—	—	2.93
Overall level	65	9.21	—	—	2.09
	75	4.60	0.84	—	2.51

	55	12.78	—	7.22	—
Onset level	65	12.22	1.11	3.33	—
	75	6.11	1.11	1.67	0.56
